# A rare presentation of appendicitis inside the femoral canal: case report and literature review

**DOI:** 10.1186/s40792-018-0552-y

**Published:** 2018-12-13

**Authors:** Bardia Bidarmaghz, Roderick Cyril Borrowdale, Kasra Raufian

**Affiliations:** Department of General surgery, Redcliffe Hospital, Redcliffe, QLD 4020 Australia

**Keywords:** De Garengeot hernia, Femoral hernia, Appendicitis

## Abstract

**Background:**

Femoral hernia accounts for 3% of all the hernias, and in 0.5–5% of cases, the appendix can migrate through the femoral hernia and is called de Garengeot hernia. It is a very rare condition, and the incidence of appendicitis in this type of hernia is as low as 0.08–0.13%.

**Case presentation:**

We bring into discussion a case of a 47-year-old female who presented to the emergency department with a painful right-sided groin lump for the past 2 days. After initial resuscitation, a CT scan was requested which showed the presence of inflamed appendix inside the femoral canal. She was taken to the operative theatre, and during the laparoscopy, the appendix was identified migrating through the femoral canal and it could not be retracted into the peritoneal cavity; therefore, the mesoappendix was divided and the operation converted to the open low approach. After identifying the femoral hernia sac and opening it, the appendix was removed and herniorrhaphy was performed. Our patient had an uneventful recovery and was discharged on the following day.

**Conclusion:**

We report a rare case of de Garengeot hernia which was diagnosed preoperatively. Because of its non-specific presentation, patients are usually diagnosed with incarcerated femoral hernia and are taken to operative theatre and the final diagnosis is made intra-operatively. Due to its rarity, there is no standard approach for this condition, and emergency appendicectomy and concurrent herniorrhaphy is the mainstay of treatment. In this paper, we present different surgical methods for the treatment of this type of hernia.

## Introduction

Hernias are usually named after the surgeons who first described them. The eponym Littre’s hernia refers to an inguinal hernia containing a Meckel’s diverticulum and was named after Alexis de Littre. Maydl’s hernia, which describes the presence of an incarcerated small intestinal loop in a “W” configuration inside a single hernia sac, was first described by Karel Maydl. The presence of the appendix inside the inguinal canal is called Amyand’s hernia after being first reported by Claudius Amyand [1].

In 1731, Rene Jacques Croissant de Garengeot (1688–1759), who was a Parisian surgeon, first described a rare case of the appendix inside the femoral canal [2] and Hevin did the first appendicectomy in an incarcerated femoral hernia in 1785 [3]. This phenomenon occurs in 0.5–5% of femoral hernias, and the presence of acute appendicitis within a de Garengoet hernia is rarer still with an incidence of 0.08–0.13% [4]. Pre-operative diagnosis of de Garengeot hernia can be challenging because of its non-specific history and clinical presentation and there are no published guidelines regarding the optimum operative management [5]. Diagnosis of this condition is usually made intra-operatively.

In this paper, we present a middle-aged female patient who presented with an inflamed appendix inside the femoral canal who underwent surgery. We also describe different surgical techniques that may be employed in the treatment of this type of hernia.

## Case presentation

**Fig. 1:**
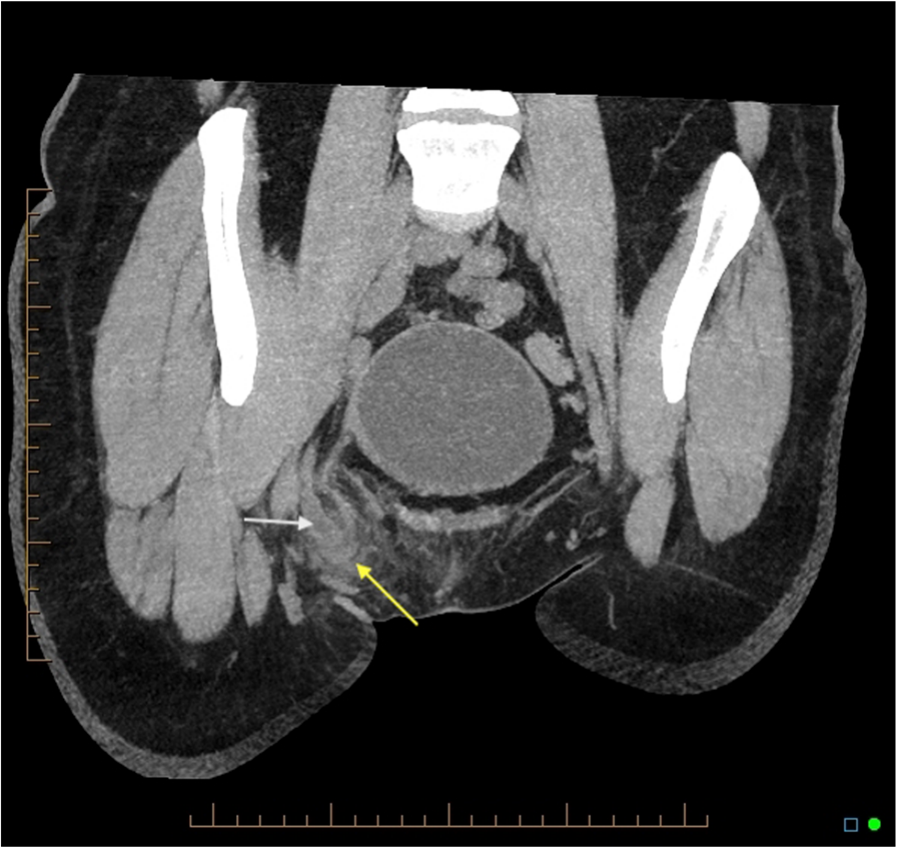
CT scan coronal view shows appendicitis (white arrow) inside the femoral canal (yellow arrow)

A 47-year-old female was referred to the emergency department by her general practitioner after noticing a lump in her right groin. She mentioned that 2 days prior to presentation that a lump appeared suddenly and was initially uncomfortable but then developed increasing pain and erythema. The patient denied nausea, vomiting, fever, or change in bowel habit. The patient did not have any past significant medical history apart from being a smoker and had a BMI of 25.6. On arrival to the ED, the patient was resuscitated, and on examination, a 3 × 3 cm erythematous, irreducible lump with minor local tenderness was identified in the right groin below the inguinal ligament. The patient had a leucocytosis of 12.1 with normal serum biochemistry. A CT scan demonstrated a blind-ended structure with surrounding fat stranding inside the femoral canal (Fig. 1). The patient was diagnosed with a de Garengeot hernia and was taken to the operating theatre.

**Fig. 2:**
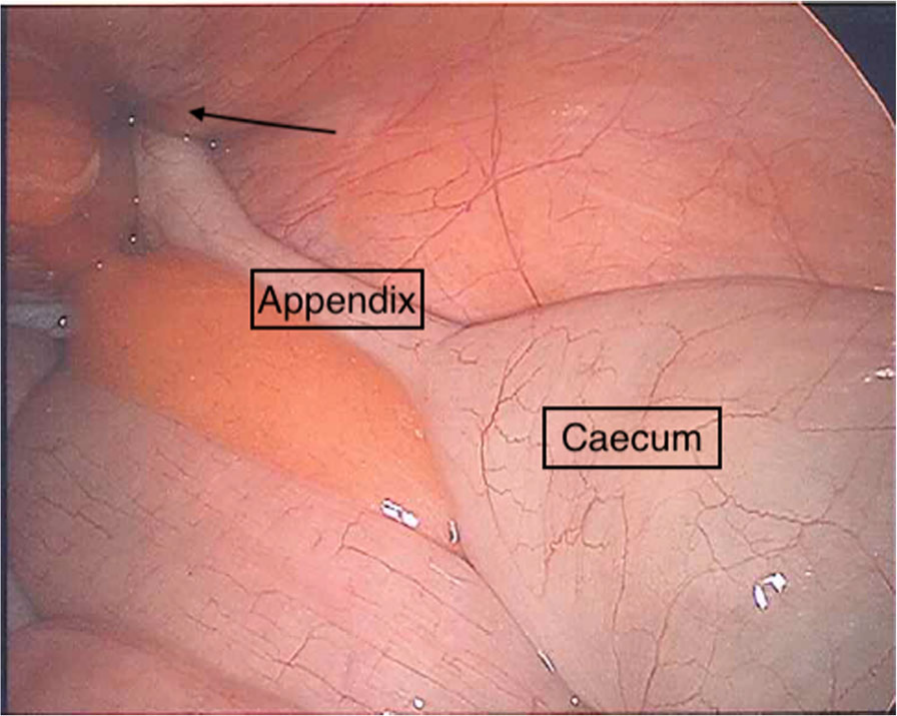
Laparoscopic view of the appendix traveling through the femoral canal

**Fig. 3:**
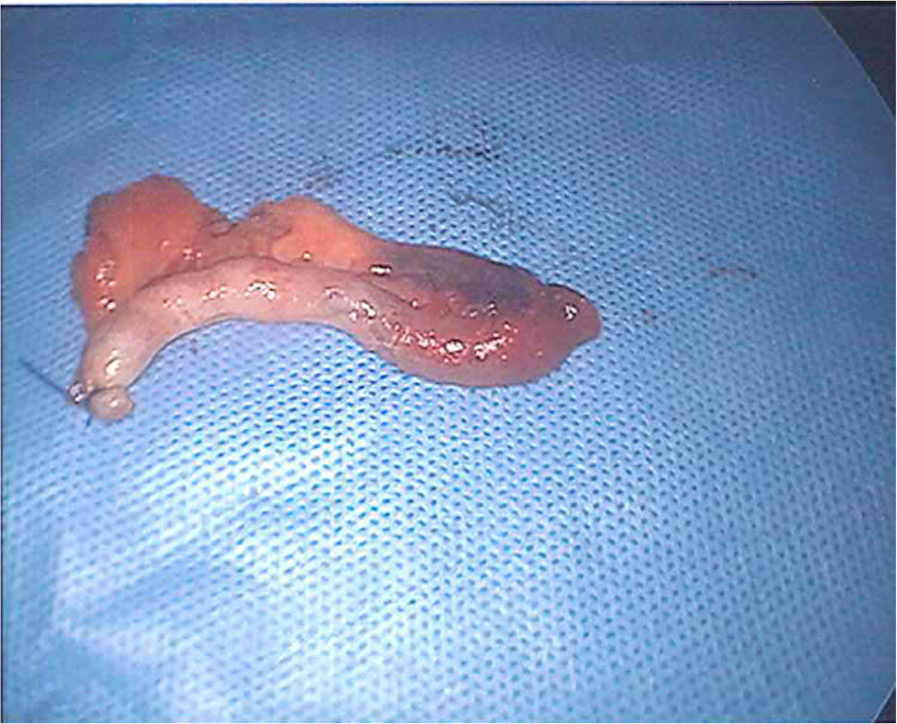
Macroscopic view of the inflamed appendix

The patient underwent laparoscopy where the appendix was found to have migrated through the femoral canal (Fig. 2), and attempts at reduction into the peritoneal cavity proved to be unsuccessful. The mesoappendix was divided, and the base of the appendix was divided between endoloops. A standard low approach incision was made over the right groin swelling, and a strangulated right femoral hernia containing an inflamed appendix with strangulated extraperitoneal necrotic fat was identified. The hernia sac was opened, and the appendix delivered and removed (Fig. 3), and after appropriate dissection, the necrotic tissue was excised and hernia sac suture ligated. Because of the presumed bacterial translocation, the femoral hernia defect was closed using interrupted non-absorbable sutures. The patient was able to be discharged the following day and underwent an uneventful recovery. The histopathology confirmed acute appendicitis.

## Discussion

Since the first description of the de Garengeot hernia in 1731, less than 100 cases have been reported in the literature [6]. Femoral hernia is more common in women and de Garengeot hernias occur in females three times more often than in males. Smoking, post-menopausal status, pregnancy-related changes, constipation, chronic cough, and older age are risk factors for de Garengeot hernia [5]. There are two main theories for the explanation of this condition. According to Zissin et al., an anatomically large caecum pushes the appendix inside the femoral canal [7], but Nguyen et al. claim that abnormal rotation of the intestine during embryologic development makes the appendix more prone to herniation through the femoral canal [8]. The sequence of an inflamed appendix inside the femoral canal is also unclear. Some authors believe that the migration of the appendix happens first and then due to the narrow, rigid neck of the femoral canal, incarceration and strangulation happens. Others, however, emphasise that appendicitis happens first followed by migration into the femoral canal [9].

Pre-operative diagnosis of de Garengeot hernia can be challenging due to its non-specific presentation. Patients usually present with a tender right-sided groin lump and after being diagnosed with an incarcerated femoral hernia are brought to the operating theatre and found to have an appendix in the femoral canal intra-operatively [7]. These patients usually do not have signs of intra-peritoneal pathology because the nature of the femoral canal tends to limit the spread of sepsis into the peritoneal cavity [10].

Radiologic findings are non-specific. Although ultrasound is frequently used for detection of groin herniae, it is operator-dependent and only two cases have been reported that were diagnosed by ultrasound pre-operatively [11]. MRI should be reserved for young pregnant women [12], and CT scan is the modality of choice in the diagnosis of de Garengeot hernia as it will show a tubular structure projecting below the caecum and into the femoral canal with adjacent fat stranding [13].

Emergency appendicectomy and femoral herniorrhaphy is the treatment of de Garengeot hernia. Due to the rarity of this condition and its typically intra-operative diagnosis, there is no standard surgical approach for this condition. Laparoscopic and open approaches have been advocated in the literature. Beysens et al. first described laparoscopic appendicectomy with totally extraperitoneal repair of the femoral hernia in 2013 [14], which has the advantage of shorter hospital stay and reduced post-operative pain. Alternate surgical approaches include the McEvedy incision which provides superior visualisation and access when strangulation is suspected [5]. With the infra-inguinal (Lockwood Low) approach, which is the preferred method in elective repair, an oblique incision is made parallel to the inguinal ligament. This approach has the disadvantage of limited access to the viscera if compromised. In the trans-inguinal (Lotheissen) approach, the skin incision is made 2 cm above the inguinal ligament and the inguinal canal is exposed. This approach carries the risk of reoccurrence of a hernia which can be difficult to repair. In 1994, Sorelli et al. proposed the King’s College an approach that involves a single incision through which all the above open methods can be undertaken. Through an inguinal incision 1 cm above the medial half of the inguinal ligament, dissection is made down to the external oblique aponeurosis and superior and inferior flaps are fashioned. This method provides a possibility of differentiating between inguinal and femoral herniae, and if a femoral hernia contains compromised bowel, the rectus sheath is divided along the linea semilunaris 4 cm above the inguinal ligament. After exposing and medially retracting the rectus abdominus muscle, the transversalis fascia and peritoneum are divided and access is made into the peritoneal cavity [15].

The major post-operative complication of de Garengeot hernia repair is wound infection which occurs in 14 to 29% of cases [3]. Delay in diagnosis, older age, poor nutritional status, and involvement of multiple tissue planes in repairing the hernia all increase the risk of post-operative infection [8]. In our patient, after laparoscopically dividing the appendix and following the King’s College approach, the external oblique fascia was exposed and the inferior flap mobilised, and through the Lockwood Low approach, the appendix was removed and femoral herniorrhaphy performed.

## Conclusion

De Garengeot hernia is a rare condition, and clinicians should have a high index of suspicion when they encounter a patient with right-sided incarcerated or strangulated femoral hernia. CT scan is the modality of choice for diagnosis but most cases are discovered intra-operatively. The treatment of this type of hernia is emergency appendicectomy and concurrent herniorrhaphy.

## Abbreviations

BMI: Body mass index
CT: Computed tomography
ED: Emergency department
